# Advanced Fitting Method for the Kinetic Analysis of Thermogravimetric Data

**DOI:** 10.3390/molecules28010424

**Published:** 2023-01-03

**Authors:** Ivan Bondarchuk, Sergey Bondarchuk, Alexander Vorozhtsov, Alexander Zhukov

**Affiliations:** 1 Laboratory for High Energy and Special Materials National Research, Tomsk State University, Lenin Avenue, 36, 634050 Tomsk, Russia; 2 Faculty of Biology and Chemistry, Tomsk State Pedagogical University, Kievskaya Str., 60, 634061 Tomsk, Russia; 3 Center for Additive Technologies National Research Tomsk State University, Lenin Avenue, 36, 634050 Tomsk, Russia

**Keywords:** thermogravimetric analysis, kinetic triplet, modified Arrhenius equation, general temperature integral, fitting method, solid-state reaction mechanism, kinetic analysis

## Abstract

The article considered the solution of the inverse problem of chemical kinetics of the analysis of experimental data of a thermogravimetric experiment at a constant sample heating rate. The fitting method for identifying the parameters of a kinetic triplet using the integral method for a model of a solid-state reaction based on the modified Arrhenius equation is described. The effectiveness of the proposed approach was confirmed by solving test cases for low, medium, and high rates of material conversion. Unlike other methods, setting the parameters of the reaction mechanism is not required, as they are determined by the solution. Solutions for real data of TGA studies with high and low sample heating rates were compared with the results obtained by other authors and experimental data. A description of the full cycle of calculations used to identify kinetic parameters from thermogravimetric experimental data is given, from the derivation of calculated relationships to the implementation of a short (three to five formulas) program code for MS Excel spreadsheets. The presented code is easy to verify and reproduce and can be modified to solve various problems.

## 1. Introduction

Thermogravimetric analysis (TGA) with a constant heating rate applied to the sample is widely used to identify the kinetic parameters of solid-state reactions using experimental data. A TGA experiment determines the change (conversion) in mass of a sample measured as a function of time (or temperature).

Two main classes of methods are used to analyze the kinetic data of solid-state reactions. The first class includes the so-called fitting methods which are used for experimentally obtained single dependence of mass change in the sample on time t at a specific heating rate β, while the second class consists of model-free (isoconversion) methods that require several dependences with different β values [1,2,3,4,5,6,7,8,9,10,11,12,13].

A differential kinetic equation is often used to describe the process of transformation (decomposition) of a substance. This equation relates the rate of a solid-state reaction dα/dt of change in the dimensionless degree of conversion α with a given function of the reaction mechanism f(α) and with the Arrhenius reaction-rate constant k(T). For example, the assumption of a first-order reaction in the Kissinger method, which is widely used, may lead to large evaluation errors in TGA studies applying temperature programs other than linear heating [1,2,14].

The mechanism function of a reaction f(α) can be determined using the logical choice method [6,15], some specific methods [7,11], or using one of 40 model functions [12,16,17]. The criterion is the maximum value of the correlation coefficient approximating the dependencies used in a particular method. Prior to kinetic analysis, the thermal decomposition reaction mechanism f(α) is assumed to have a specific mathematical form.

Fitting methods are used under the assumption that the activation energy E is constant for the entire range of conversion, whereas isoconversion methods provide a significantly more insightful alternative by considering the variability of E(α) [2]. Nevertheless, fitting methods are widely used and are constantly being improved [2] in terms of the accuracy of determining model parameters. It should be noted that for practical use the dependence E(α) obtained using the isoconversion method is included as an average value E = const in the final form of the mathematical model of the reaction [6].

A significant difficulty in implementing both fitting and isoconversion methods is the lack of a reliable criterion for choosing an adequate model of the reaction mechanism f(α), corresponding to the actual process [18]. In most fitting methods, a dependence f(α) is initially specified. In model-free isoconversion methods, the pre-exponential A of the Arrhenius function is determined using a given reaction mechanism f(α) and an obtained apparent activation energy value [6,19].

The classical Arrhenius equation is widely used for calculations in both methods, which may be considered as a disadvantage, since a number of studies have shown that its various modifications significantly improve the accuracy of determining the kinetic parameters [20,21]. In particular, Criado et al. [20] examined the dependence of the preexponential factor on temperature and estimated the errors involved in the activation energy calculated from isothermal and non-isothermal methods. The study demonstrated that the error in determining the activation energy calculated ignoring the dependence of A on temperature T can be rather large and depends on the parameter x=E/RT (R is the universal gas constant), independent of the experimental method used.

The general differential equation of the model, which is widely used for thermal experiments, does not have any limitations regarding the temperature effect (heating or cooling the sample) [12]. Upon cooling, single-step thermally stimulated reactions are usually studied using the data obtained under continuous heating run [21], as it is assumed that the kinetics measured on cooling is similar to that measured on heating. When studying such processes, a number of authors concluded that the modified Arrhenius equation provides higher accuracy of the results. Some observed differences in the kinetics measured on heating and cooling can be reduced by changing the curvature of the Arrhenius plots [22]. Moon et al. [3] when studying the mechanical properties of polymers at low temperatures noted that the use of the modified Arrhenius equation instead of the general one reduced the average deviation between the calculated and experimental values from 42% to 17%.

The parameters of the mathematical model of the reaction (the so-called kinetic triplet A,E,f(α)) were determined by solving the inverse problem of chemical kinetics based on the obtained experimental data. The adequacy of the reaction model was estimated by the coincidence of the initial experimental data and the results of solving the direct problem of kinetics for known parameters of the process model, identified from the solution of the inverse problem of kinetics. Most often, the criterion was either the square of the difference between the calculated and experimental values of the degree of conversion, or the maximum value of the non-linear coefficient of determination $R^{2}$. Nevertheless, in a number of studies [4,7,11,16,23,24], the accuracy of solutions was confirmed by the coefficient of determination for some internal intermediate approximations obtained while solving the inverse problem. In this study, we did not consider this method to verify the results to be quite correct.

In practice, fitting methods require either numerical differentiation of experimental data (differential methods), or the integration of the general equation of the model (integral methods). The former requires smoothing of experimental tabular data, while in the latter case the difficulties consist in the calculation of non-elementary integrals. Kinetic analysis using integral methods is most often used by researchers and is widely presented in relevant studies [4,8,12,13,14,24]. Over the last 50 years, a large number of studies have discussed improving the accuracy of approximations of the temperature integral (the integral of the Arrhenius function), which does not have an analytical expression. Infinite series, rational and special functions, series of Chebyshev polynomials, and approximations obtained directly from numerical solutions are used to approximate the values of the integral [1,2,5,6,10,14,25,26,27]. At present, according to the literature, the proposed approximations and approaches associated with them are less significant, since the solution of the corresponding inverse problems of chemical kinetics is usually performed on a computer, and the exact numerical calculation of temperature integral [28] is easily included into the algorithms for solving inverse problems of kinetics.

We should also note that generally studies do not consider issues related to the discussion of the adequacy of the description of mathematical models and methods of their implementation together. Either code fragments [9] or flowcharts [10] are given.

The aim of this study was to describe the fitting method for identifying the parameters of the kinetic triplet using the integral method for a model based on the modified Arrhenius equation. The study was also to present an algorithm for the numerical calculation based on the proposed method, which:

- Does not use any approximate estimates of the temperature integral or data approximations in the solution;
- Does not require any detailed preliminary assumptions about the reaction mechanism;
- Can be easily generalized for non-linear heating regimes;
- Can be simply implemented by a short (3–5 MS Excel formulas without VBA macros) spreadsheet program code, which is easily verified and reproducible.

## 2. Results of Theoretical Analysis— Proposed Approach

The kinetics of solid-state reactions is described by a differential equation taking into account the features of the specific reaction mechanism f(α). The reaction rate constant is linked to the dimensionless degree of conversion:

(1) $$\frac{d\alpha }{dt}=k\left(T\right)\cdot f\left(\alpha \right)\;,\;\;\alpha \in \left[0;1\right].\;$$

The literature suggests using specific dependence for a formal description of the mechanism of solid-phase reactions f(α). In this case, the dependence is specific for different reactions (chemical reaction, diffusion, random nucleation and nuclei growth, phase-boundary reaction).

Information on the real mechanism of the reaction allows choosing an optimal reaction mechanism f(α). When there is no information available, a special study or justification is conducted to select the type of reaction mechanism [13].

Most studies provide a list of expressions for the function f(α). Almost all the expressions are listed, for example, in [15,29].

The temperature dependence of the rate constant k(T) (where T is the absolute temperature) is expressed by means of the modified Arrhenius equation (at q = 0 corresponding to the simple or initial form):

(2) $$k\left(T\right)=A\cdot {\left(\frac{T}{1\;K}\right)}^{q}\cdot \exp\left(-\frac{E}{RT}\right)\;,\;\;R=8.3144\;J\cdot {\mathrm{mol}}^{-1}\cdot K^{-1}\;,\;$$

where q is temperature parameter of the modified Arrhenius function (MAF);

A is pre-exponential factor (frequency factor), [A] = [t]^−1^;

E is the apparent activation energy, [E] = [RT] = J·mol^−1^.

The simple (initial) form of the Arrhenius equation was a special case with q = 0, but for some solid-state reactions q tended to range from −1.5 to 2.5, and for pyrolytic reactions or gas combustion reactions it had a wider range (from −4 to 4), as reported [26]. In particular, q = 0.5 was predicted by collision theory in the homogeneous gas phase and q = 1 was predicted by transition state theory. Other modifications of the Arrhenius equation, including the stretched exponential, super- and sub-Arrhenius models [21,30,31], can also be easily used within the proposed approach.

The general equation of the solid-state reaction is the mathematical model of the process that could be expressed by a combination of the above relationships

(3) $$\frac{d\alpha }{dt}=A\cdot {\left(\frac{T}{1\;K}\right)}^{q}\cdot \exp\left(-\frac{E}{RT}\right)f\left(\alpha \right)\;.$$

When the temperature rises at a constant rate is expressed as follows:

(4) β=dT/dt=const ,

after substitution of t by T, Equation (3) can be represented as:

(5) $$\frac{d\alpha }{dT}=\frac{A}{\beta }{\left(\frac{T}{1\;K}\right)}^{q}\cdot \exp\left(-\frac{E}{RT}\right)f\left(\alpha \right)\;,\;\;\alpha \left(T=T_{0}\right)=\alpha_{0}.$$

When the kinetic triplet A, E, f(α) is known, Equation (5) with boundary conditions with $\alpha_{0}$ at temperature $T_{0}$ determines the mathematical formulation of the direct problem of chemical kinetics (the Cauchy problem [32]) for the calculation of the dependence $\alpha \left(T\right),\;T>T_{0}$.

To identify the kinetic triplet, Equation (5) was also used when the inverse problem of chemical kinetics was solved based on the results of the TGA experiment, the given data table $\left\{T_{i},\alpha_{i}\right\}\;i$ = 0, 1, …, N.

According to the physical meaning, the analyzed data should start with the value $\alpha_{0}$ = 0. However, when the sample mass was small (only a few milligrams), the analytical signal at the initial part of the experimental curve α(T) can be comparable to the instrumental error, so data processing was usually carried out starting from values $\alpha_{0}$ > 0 ($\alpha_{0}$ ≈ 0.01 − 0.1).

The function of the process f(α) depends on the reaction mechanism, which can be expressed by the Šesták–Berggren equation [18]:

(6) $$f\left(\alpha \right)=\alpha^{m}{\left(1-\alpha \right)}^{n}{\left[-\ln\left(1-\alpha \right)\right]}^{p}\;,$$

where: m, n, and p are empirically obtained exponent factors, one of which is always zero [18]. The combinations of different orders of m, n, and p make it possible to predict probable mechanisms.

It should be noted that the algorithm for the identification of the required parameters A,E,f(α) of the kinetic triplet is difficult to implement, when the Šesták–Berggren function (6) is included in Equation (5) of the process. In most cases, a specific simplified form of the function f(α) is chosen [12,29].

When the integral method is implemented to solve the inverse problem of kinetics, the variables are separated in Equation (5) and the r.h.s. and l.h.s. are integrated:

(7) $$\overset{\alpha }{\underset{\alpha_{0}}{\int }}\frac{d\alpha }{f\left(\alpha \right)}=\frac{A}{\beta }\overset{T}{\underset{T_{0}}{\int }}{\left(\frac{T}{1\;K}\right)}^{q}\cdot \exp\left(-\frac{E}{RT}\right)dT\;,$$

where l.h.s is the so-called integral form of the conversion function, equal to:

(8) $$g\left(\alpha_{0},\alpha \right)=I^{\alpha }\left(\alpha_{0},\alpha \right)=\overset{\alpha }{\underset{\alpha_{0}}{\int }}\frac{d\alpha }{f\left(\alpha \right)}=\overset{\alpha }{\underset{\alpha_{0}}{\int }}\frac{d\alpha }{\alpha^{m}{\left(1-\alpha \right)}^{n}{\left[-\ln\left(1-\alpha \right)\right]}^{p}}\;.\;$$

When $\alpha_{0}$ ≠ 0 the time corresponding to the temperature changes at $0\leq \alpha \leq \alpha_{0}$ can be attributed to the induction period.

The integral of the r.h.s. of Equation (7) is non-elementary and cannot be determined analytically. A number of studies that estimated the value of this temperature integral proposed different techniques and approximations and included many references (e.g., [1,2,5,6,10,14,25,26,27]).

The Coats–Redfern approximation by with parameter q = 0 is the most widely used approximation of the temperature integral [5]

(9) $$I^{T}\left(T_{1},T_{2}\right)=\overset{T_{2}}{\underset{T_{1}}{\int }}{\left(\frac{T}{1\;K}\right)}^{q}\cdot \exp\left(-\frac{E}{RT}\right)dT\;,\;$$

when q ≠ 0 the previous relationship is as follows:

(10) $$I^{T}\left(0,T\right)\approx \frac{RT^{2}}{E}{\left(\frac{T}{1K}\right)}^{q}\cdot \exp\left(-\frac{E}{RT}\right)\cdot \left[1-\left(q+2\right)\frac{RT}{E}\left(1-\left(q+3\right)\frac{RT}{E}\right)\right]\;.\;$$

According to the proposed approach, a numerical method is used to calculate the temperature integral.

Non-isothermal thermogravimetric analysis with a linear temperature rise at a constant heating rate β = const is a standard method for the kinetic study of solid-state reactions which implies monitoring the mass change of the tested sample as a function of time or temperature. Using Equation (4), the initial data of the thermogravimetric experiment can be transformed into the data table $\left\{T_{i},\alpha_{i}\right\}\;i$ = 0, 1, …, N.

In accordance with Equation (7), it is possible to calculate a set of “constants” $A_{\beta i}$ of the frequency factor A for each range $\left[T_{0}-T_{i}\right]$ and $\left[\alpha_{0}-\alpha_{i}\right]$ (i = 1, 2, …, N) of the initial data table $\left\{T_{i},\alpha_{i}\right\}$

(11) $$A_{\beta i}=I^{\alpha }\left(\alpha_{0},\alpha_{i}\right)/I^{T}\left(T_{0},T_{i}\right)\;,\;\;i=1,\;2,\ldots ,\;N\;.\;$$

Within the proposed approach, it is assumed that the optimal values of the kinetic triplet A, E,(m,n,p) are reached when the set of calculated constants $A_{\beta i}$ (i = 1, 2,…, N) has a minimum scatter since ratio A/β is constant by definition. This assumption can be mathematically expressed by the functional ℱ, which corresponds to the variation coefficient [8]:

(12) $$ℱ=\frac{1}{\bar{A_{\beta }}}\cdot \sqrt{\frac{\sum_{i}{\left(A_{\beta i}-\bar{A_{\beta }}\right)}^{2}}{N}\;},\;\;\bar{A_{\beta }}=\frac{1}{N}\underset{i}{\sum }A_{\beta i}\;.$$

In other words, the optimal parameters E and m,n,p should be selected in such a way so that there is minimal difference between total deviation of the constants $A_{\beta i}$ (i = 1, …, N) calculated by formula (12) and the average $\bar{A_{\beta }}$. Due to the minimization of the functional (12), the obtained values of E,(m,n,p) and $A_{\beta }=A/\beta =\bar{A_{\beta }}$ determine the optimal parameters of the kinetic triplet. The undoubted advantage of the proposed approach is that in the process of searching for the optimal values of A, E,(m,n,p), the number of parameters that vary during optimization is reduced by one.

## 3. Verification of Method

**Figure 1:**
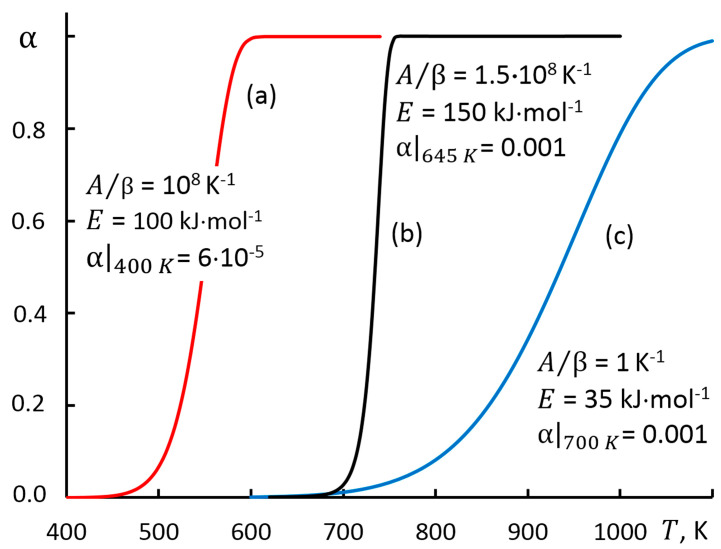
Simulation curves α(T) for test cases.

The accuracy of the calculations made within the proposed approach is ensured by the MS Excel program code and was tested on theoretical simulation curves at various linear heating rate β, reaction mechanisms f(α) and temperature range with varying degrees of conversion 0.1 < α < 0.9 (Figure 1). The parameters of the selected test cases are characterized by the width of the temperature range, which determines the low, medium, and high degrees of the conversion rate.

According to the generally accepted classification [4,29], the models are indexed by symbols A_2_ and R_1_, and their parameters are given in Table 1. The dependences α(T) were obtained by solving the direct problem of kinetics and were chosen as test problems: presented models (a) and (c) were analyzed in the study [4]; the modified Arrhenius equation was used in model (b) which was characterized by a narrow conversion temperature range [6].

The value of the complex x=E/(RT) [4,28] has a significant impact on the accuracy of the approximation of the Arrhenius integral. In the case of Figure 1c, variation interval is 3.8 < x < 6.0, and for Figure 1a it is 20 < x < 30 (Table 1).

Perez–Maqueda et al. [4] analyzed kinetic curves and concluded that the Coats–Redfern method showed the best accuracy in identifying the activation energy. In this study, we compare the proposed approach with the Coats–Redfern method. The numerical calculation of the general temperature integral is one of the main aspects determining the accuracy of the proposed approach.

Table 2 presents the comparison of the relative errors of direct numerical calculation and the Coats-Redfern approximation in the range of values of complex x, which is interesting on practical grounds, when integrated within 500–600 K. The relative error was estimated by numerical integration value with an accuracy of 10^−^^10^ according to the Simpson’s rule [32].

The aim of the study by Perez–Maqueda et al. [4] was to analyze the errors of various approximations used in the algorithms and not to conduct a kinetic analysis.

Moreover, as emphasized by the authors [4], the reaction mechanism could not be determined adequately based on a single curve α(T), that is why the authors [4] used specific types of reaction mechanisms coded by symbols A_2_ and R_1_ for the formal kinetic description.

To evaluate the efficiency of the proposed approach, we extended the list of problems to be solved. It is assumed that indexes of power n,p in the Šesták–Berggren function (6) are unknown in advance, except m = 0, i.e., $f\left(\alpha \right)={\left(1-\alpha \right)}^{n}{\left[-\ln\left(1-\alpha \right)\right]}^{p}$, where n and p are required parameters and their values should be determined.

The parameter q of the modified Arrhenius Equation (5) is considered to be given.

The value of the general temperature integral of the r.h.s. Equation (7) is calculated according to the midpoint rule [32] for 10,000 integration subintervals

(13) $$I^{T}\left(T_{1},T_{2}\right)=\overset{T_{2}}{\underset{T_{1}}{\int }}{\left(\frac{T}{1\;K}\right)}^{q}\cdot \exp\left(-\frac{E}{R\cdot T}\right)dT\approx h_{T}\overset{10000}{\underset{j=1}{\sum }}{\left(\frac{T_{j}}{1\;K}\right)}^{q}\cdot \exp\left(\frac{E}{R\cdot T_{j}}\right)\;,$$

$$T_{j}=T_{1}+\left(j-0.5\right)h_{T},\;j=1,2,\ldots ,10000\;,\;h_{T}=\left(T_{2}-T_{1}\right)/10000\;.$$

The necessary sequence of numbers (1, 2, …, 10000) to calculate the general temperature integral in MS Excel is generated using the array formula or CSE formula (F2, Ctrl + Shift + Enter) [33] of the ROW(A1:A10000) function. This algorithm is more detailed in [28].

A numerical integration to calculate of the l.h.s. by Equation (7) was used, such as in Equation (13).

(14) $$g\left(\alpha_{1},\alpha_{2}\right)=I^{\alpha }\left(\alpha_{1},\alpha_{2}\right)=\overset{\alpha_{2}}{\underset{\alpha_{1}}{\int }}\frac{d\alpha }{\alpha^{m}{\left(1-\alpha \right)}^{n}{\left[-\ln\left(1-\alpha \right)\right]}^{p}}\approx h_{\alpha }\overset{10000}{\underset{j=1}{\sum }}\frac{1}{\alpha_{j}^{m}{\left(1-\alpha_{j}\right)}^{n}{\left[-\ln\left(1-\alpha_{j}\right)\right]}^{p}}\;,$$

where $\alpha_{j}=\alpha_{1}+\left(j-0.5\right)h_{\alpha },j=1,2,\ldots ,10000\;,h_{\alpha }=\left(\alpha_{2}-\alpha_{1}\right)/10000$

Kinetic Šesták–Berggren Equation (6), containing three exponential terms, can describe any TGA curve [34]. Nevertheless, it is noted that the mathematical analysis of Equation (6) showed [35] that no more than two kinetic exponents are needed. Therefore, one of the exponents of relationship (6) can be excluded, and, in particular, as is done below, the mechanism (6) of the reaction can be identified at m = 0.

Algorithm to solve the inverse kinetics problem for the test curve Figure 1a involves the following steps.

**Figure 2:**
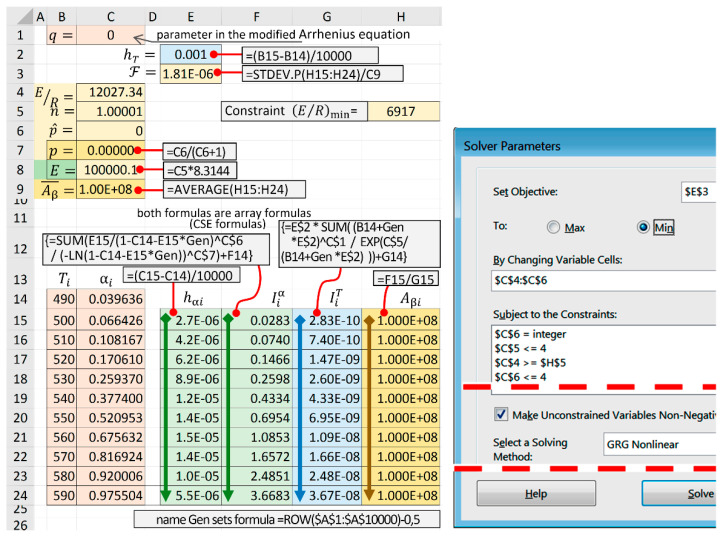
MS Excel worksheet screenshot (after executed the Solver add-in) and the Solver Parameters panel.

1. Generated array of initial data $\left\{T_{i},\alpha_{i}\right\}$ of the test example (Figure 1 and Table 1*)* is shown in the screenshot Figure 2, in the cell range B14:C24. The parameter q of the general temperature integral was entered in cell C1, and in this case it was equal to zero. The ROW($A$1:$A$10000)−0.5 formula in Excel was used to create the Excel Gen name via Formulas → Define Name.

2. The minimum allowable value of E/R was entered into cells C4 and H5. In this test case, this value was 6917. The method of its calculation is given below when analyzing the uniqueness of the search for the minimum of the functional. Cells C6 andC7 contain the maximum values of the parameters of the truncated Šesták–Berggren equation (6), which, in accordance with their physical interpretation [14,18,29], vary in the ranges 0 ≤ m ≤ 4 and 0 ≤ $\hat{p}$ ≤ 4; the exponent was determined by integer values $\hat{p}$ = 0, 1, 2, 3, 4 –i.e., p = 0, 1/2, 2/3, 3/4, 4/5. Here, the values were set to 4 and 4, respectively.

After executing the Solver add-in [8] in cells C8, C5, and C7, the optimal values of E, m, and p were calculated.

3. The integration step of the general temperature integral was calculated in cell F2 for the temperature range $T\in \left[T_{i};T_{i+1}\right]$, which was equal for all subintervals in this case.

The subintervals were determined in cell E15 to calculate the integral $I_{i}^{\alpha }=g\left(\alpha_{i},\alpha_{i+1}\right)$ in cell F15 using Equation (14) for the current values of $\alpha \in \left[\alpha_{i};\alpha_{i+1}\right]$. The formula to calculate the general temperature integral according to Equation (13) was entered into cell G15. Excel formulas in cells F15 and G15 are used as CSE array formulas [33]. Based on the ratio of the integrals $I_{i}^{\alpha }$ and $I_{i}^{T}$, the value of the pre-exponential factor $A_{\beta i}$ for the given interval was determined in H15 according to Equation (11). The formulas in E15:H15 were copied to line 24 by dragging the fill handle in Excel [36].

4. The average (=AVERAGE(·)) value $\bar{A_{\beta }}$ was determined in C9, according to Equation (12), the functional ℱ was calculated in F3 by using the STDEV.P(·) function in Excel.

5. When running the Solver add-in, the Solver Parameters panel was displayed. The basic parameters for the minimization of the functional ℱ were set using the generalized reduced gradient algorithm (“GRG Nonlinear” are set (Figure 2, right side).

To increase the rate and ensure convergence, the appropriate restrictions on the variable parameters are described in the Subject to the constraints box. In Options, we set advanced settings of the solution:

– The fields “Restriction accuracy” and “Convergence” set to 0.0000001;
– The field “Integer optimality, %” set to 0;
– The “Derivatives” radio button fixed in the “Central” position;
– The flag “Use automatic scaling” sets.

Optimization was run by clicking the Solve button on the main Solver panel.

The screenshot of the worksheet after Solver executed optimization is shown in Figure 2, left side. The “true” values of the mechanism parameters (6), activation energy E and pre-exponential factor A according to Equation (5) of the reaction model are shown in cells C5 and C7–C9.

If necessary, the final values of parameters n and p with fewer fractional digits could be adjusted, which should be followed by restarting the Solver when there was only one E/R variable (cell C4).

For Windows 7 (Home Edition) and MS Office Excel 2016 (Intel^®^ Core™ i7-3770 processor; 3.40 GHz), the solution time was ≈ 17 s.

**Figure 3:**
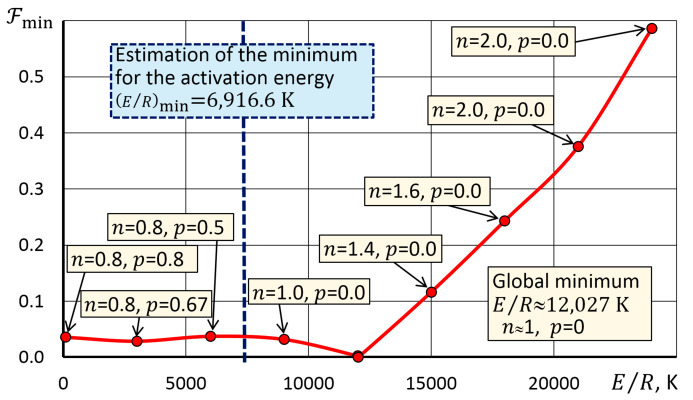
Dependence of the minimum value of the functional on the activation energy.

The uniqueness of the search for the minimum of the functional (12) can be confirmed by the diagram in Figure 3. The diagram for a number of E/R values showed the optimal exponents n and p of the Shestak–Berggren dependence, providing the maximum agreement between the solution and the experimental curve α(T). It can be seen from the diagram that at small E/R, there are local minima in front of the global minimum. One of the effective strategies for finding minimum for the Solver algorithm in this case was to set the initial ${\left(E/R\right)}_{\min}$ value on a curve segment that decreased from the left to a minimum (Figure 3).

**Figure 4:**
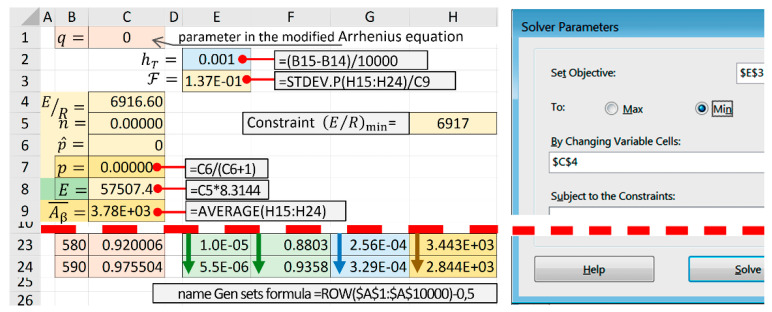
MS Excel worksheet screenshot (after executed the Solver add-in) and the Solver Parameters panel.

Estimation ${\left(E/R\right)}_{\min}$ was carried out based on the assumption that this value determines the minimum level of activation energy that ensures the reaction without thermally stimulated acceleration by the f(α) mechanism, i.e., through the solution of the inverse problem of kinetics for Equation (5) with f(α) = 1. To solve this problem, a template in Figure 2 was used, where we entered zero in cells C5 and C6, and cell C4 was the only one variable parameter (Figure 4). The obtained value ${\left(E/R\right)}_{\min}$ was used as a constraint in the search for a solution in the algorithm which was used to identify a kinetic triplet using a template in Figure 2.

The other problems of the test cases were solved using the template, as shown in Figure 2 and Figure 3, where the initial data were replaced and the corresponding cell ranges in formulas E3 and C9 were corrected. For all test cases, the comparative accuracy of determining the activation energy of the proposed approach is given in Table 3.

A significant decrease in accuracy of most approximations in this range of the complex x [4,8,27] led to higher relative errors at small values x < 6 when the activation energy was determined, while no such effect was obviously noted for numerical integration when the accuracy even increased. It should be noted that when real experimental data of thermogravimetric analysis were processed at the beginning of the process, the average signal level was comparable with the background level and resolution of the measuring equipment, the initial part of the curve was excluded from the analysis. In this case, the moment of time (or the temperature corresponding to this moment), when the signal level was higher than the amplitude of the average background level, can be assumed as the induction period end.

The end of the induction period can be characterized by the beginning moment of the active stage of the process when the rate and/or the reacted sample mass is higher than the defined value (e.g., 5% of the maximum reaction rate; 2% weight loss of the initial mass of the sample, etc.).

Since in the model according to Equation (5), the reaction rate at α < 1 at T > 0 K was always different from zero. For example, the $\alpha_{ind}$ = 10^−6^ can be taken as the starting point of the induction period.

If kinetic parameters and measured values $\alpha_{0}\left(T_{0}\right)$ were known, the temperature coordinate $T_{ind}$ of the beginning moment and the length $t_{ind}$ of the induction period can be determined from the relationship:

(15) $$\overset{\alpha_{0}}{\underset{\alpha_{ind}}{\int }}\frac{d\alpha }{f\left(\alpha \right)}=\frac{A}{\beta }\overset{T_{0}}{\underset{T_{ind}}{\int }}\exp\left(-\frac{E}{RT}\right)dT,\;\;t_{ind}=\left(T_{0}-T_{ind}\right)/\beta \;.$$

It is quite easy to calculate the induction period by the Solver add-in using Equations (13)–(15), for which $T_{ind}$ is the variable parameter, but the objective function was the minimization of the difference between the right and left-hand sides of Equation (15).

In particular, for dependence (a) Table 1 $t_{ind}$ ≈ 9.8 min, for (b) $t_{ind}$ ≈ 14.7 min and for dependence (c) $t_{ind}$ ≈ 26.5 min.

After checking the accuracy of the proposed approach on the test curves (Figure 1), it is advisable to check it on real data of the TGA experiment and compare it with the results of the solutions of other authors. A well-studied reaction [12,37] of BaCO_3_ decomposition for the classical Arrhenius equation was compared with the results of the study of the kinetics of decomposition of the energetic material HNIW using the integral method based on the Kooij formula for the modified Arrhenius equation [6]. Experiments performed with significantly different sample heating rates were selected for verification.

In this regard, the errors were compared (maximum and mean) between the results of the solutions of the direct problem (Equation (5) with initial data $\alpha_{0}\left(T_{0}\right)$) for the kinetic triplets, which were identified by different integral methods, with the initial experimental data.

The coefficient of determination as the proportion of variance in the model error was used to assess the quality of conformance of the kinetic curves. Such checking procedure of the methods adequacy corresponds to ICTAC Kinetics Committee recommendations to perform kinetic computations on thermal analysis data [13].

The modified Arrhenius equation was compared with a single curve at β = 20 K min^−1^ from an isoconversional non-isothermal experiment [6], as a result of which the following form of Equation (5) was established:

(16) $$\frac{d\alpha }{dT}=\frac{1.69\cdot 10^{14}}{\beta }{\left(\frac{T}{1\;K}\right)}^{0.5}\cdot \exp\left(-\frac{152737}{RT}\right)\cdot f\left(\alpha \right)\;,\;\;\alpha \left(T_{0}=520.5\;K\right)=0.075\;,$$

in which $f\left(\alpha \right)=\left(1-\alpha \right)\cdot {\left[-\ln\left(1-\alpha \right)\right]}^{2/3}$ was determined by a logical choice method [6,7,15].

**Figure 5:**
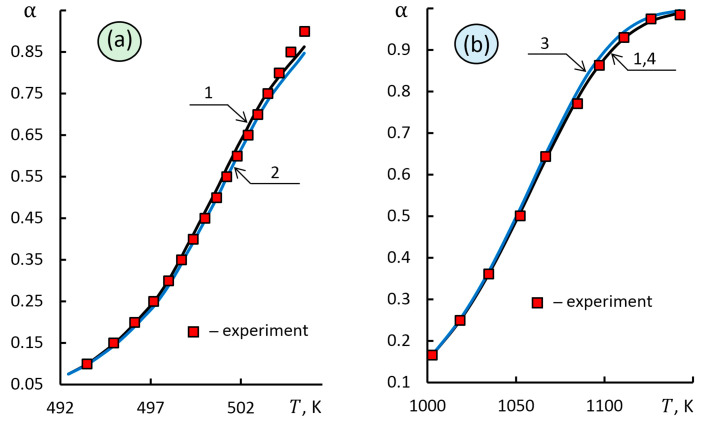
Results of solving the direct problem for kinetic triplets identified using various integral methods: **(a)** 1—proposed approach, 2—estimation [6]; **(b)** 1—proposed approach, 3—combined kinetic analysis [12,37], 4—Coats-Redfern method [5].

The results of comparing these methods (Figure 5a and Table 4) showed a difference in the calculated values of the activation energy by 5.5% with a higher accuracy of the proposed approach relative to the approximation of experimental data.

The study [12] discussed the modification of the combined kinetic analysis with an empirical equation without any preliminary assumptions about the reaction mechanism. The method proposed is based on the condition that the kinetic triplet must correspond to the general differential equation of the reaction model, regardless of the experimental conditions. The authors [12] tested this approach by analyzing TGA data on the thermal decomposition of a barium carbonate sample.

The calculation results by the proposed approach, the Coats–Redfern method [5] and the combined kinetic analysis [12] are shown in Figure 5b for kinetic data of the thermal decomposition of BaCO_3_ in vacuum at a constant decomposition rate, β = 0.2 K min^−1^. This reaction was well studied and described [12,37]. The kinetic data of the thermal decomposition of BaCO_3_ fully correspond to the F_1_ kinetic model shown in Figure 5b and presented in Table 4.

Thus, the article presented the results of verification of the proposed method using real experimental data, compared them with data obtained in other studies, which corresponded with higher accuracy of approximation of the initial data.

## 4. Conclusions

1. An integral method to estimate kinetic parameters and mechanism function of a reaction using the modified Arrhenius equation was proposed. An original statistical functional was used to solve the inverse problem for identification of optimal kinetic triplet parameters, which allowed reducing the number of variable parameters when the data fitting was conducted. The articles also presented a detailed computational algorithm to determine the induction period.

2. The effectiveness of the proposed approach was confirmed by solving several test cases for low, medium, and high conversion rates of materials. Unlike other methods, setting the reaction mechanism parameters was not required, since they were determined as the solution. Real data of TGA studies involving samples with high and low heating rates were compared with the data obtained by other authors and experimental data. In all cases, the proposed approach showed higher accuracy when determining the kinetic triplet parameters compared to methods using approximations of the generalized temperature integral for the entire range of the parameter E/(RT).

3. The article describes the full cycle of calculations made to identify kinetic parameters for the thermogravimetric experimental data—from the derivation of the calculation ratios in parametric form to the implementation of a short (three to five formulas) program code for MS Excel spreadsheet. The simplicity, brevity, and transparency of the code, which is easily accessible to researchers without programming skills, make it possible to use the code for verification and reproduction, and also provide the possibility to modify the code to solve various problems.

## Figures and Tables

**Table 1:** Tested mathematical models (Figure 1, m = 0 and n = 1).

Figure 1 Curve	Mechanism by Equation (6)	Model
(a)	$\mathrm{symbol}\;R_{1},\;p=0$	$\frac{d\alpha }{dT}=\frac{A}{\beta }\cdot \exp\left(-\frac{E}{RT}\right)\cdot \left(1-\alpha \right)\;$
(b)	symbol A_2_ for MAF, p=2/3	$\frac{d\alpha }{dT}=\frac{A}{\beta }\cdot {\left(\frac{T}{1\;K}\right)}^{0.5}\cdot \exp\left(-\frac{E}{RT}\right)\cdot \left(1-\alpha \right)\cdot {\left[-\ln\left(1-\alpha \right)\right]}^{2/3}$
(c)	$\mathrm{symbol}\;A_{2},\;p=1/2$	$\frac{d\alpha }{dT}=\frac{A}{\beta }\cdot \exp\left(-\frac{E}{RT}\right)\cdot \left(1-\alpha \right)\cdot {\left[-\ln\left(1-\alpha \right)\right]}^{1/2}$

**Table 2:** Relative errors of Arrhenius integral approximations, %.

E/R, K	2000	4000	8000	16,000	32,000	64,000
x=E/(RT)	**3.3 ÷ 4.0**	**6.7 ÷ 8.0**	**13.3 ÷ 16.0**	**26.7 ÷ 32.0**	**53.3 ÷ 64.0**	**106 ÷ 128**
Coats-Redfern method	46.4%	11.8%	3.0%	0.8%	0.2%	0.05%
proposed approach	$8\cdot 10^{-9}$%	$5\cdot 10^{-8}$%	$2\cdot 10^{-7}$%	$9\cdot 10^{-7}$%	$3\cdot 10^{-6}$%	$1\cdot 10^{-5}$%

**Table 3:** Percentage error ℰ in determining the activation energy E

Reaction	Ranges x and T,K	Method	E, J·mol	Error ℰ, %
A_2_	700 < T < 1100 $3.8<x<6,\;\bar{x}\approx 5$	Coats-Redfern	33,450	4.4
		proposed approach	35,000.7	0.002
F_1_, R_1_	400 < T < 600 $20<x<30,\;\bar{x}\approx 2.25$	Coats-Redfern	99,650	0.35
		proposed approach	10,0001.1	0.001
MAF	705 < T < 750 $24<x<26,\;\bar{x}\approx 25$	Coats-Redfern	142,032.4	2.1
		proposed approach	149,997.1	0.002

**Table 4:** Percentage accuracy of various kinetic triplets for the model Equation (5).

No	Method	n	A, min^−1^	E, J·mol	$R^{2}$	${ℰ}_{max},\%$	${ℰ}_{aver},\%$
Experiment [6], β = 20 K·$\min^{-1},\;f\left(\alpha \right)=\left(1-\alpha \right){\left[-\ln\left(1-\alpha \right)\right]}^{2/3}$							
1	proposed approach		2.56·10^13^	144,300	0.9967	3.79	2.05
2	estimation [6]		1.69·10^14^	152,737	0.9948	5.79	2.85
Experiment [12,37], β = 0.2 K·min^−1^							
1	proposed approach	1.172	7.1709·10^8^	226,630	0.9996	1.47	0.58
3	combined analysis [12,37]	1.081 * 0.0246	5.76·10^8^	225,000	0.9980	3.66	1.51
4	Coats-Redfern [5]	1.207	1.5079·10^9^	232,840	0.9996	1.65	0.59

* combined analysis results were obtained in [12] for $f\left(\alpha \right)=\alpha^{-0.0246}{\left(1-\alpha \right)}^{1.081}$

## Data Availability

Not applicable.
